# Reversible Photo-Induced Reshaping of Imprinted Microstructures Using a Low Molecular Azo Dye

**DOI:** 10.3390/polym14030586

**Published:** 2022-01-31

**Authors:** Burhan Kaban, Sekvan Bagatur, Marcus Soter, Hartmut Hillmer, Thomas Fuhrmann-Lieker

**Affiliations:** 1 Institute of Nanostructure Technologies and Analytics (INA) and Center for Interdisciplinary Nanostructure Science and Technology (CINSaT), University of Kassel, Heinrich-Plett-Straße 40, 34132 Kassel, Germany; kaban@ina.uni-kassel.de (B.K.); uk033984@student.uni-kassel.de (M.S.); hillmer@ina.uni-kassel.de (H.H.); 2 Physical Chemistry of Nanomaterials, Institute of Chemistry and Center for Interdisciplinary Nanostructure Science and Technology (CINSaT), University of Kassel, Heinrich-Plett-Straße 40, 34132 Kassel, Germany; sbagatur@uni-kassel.de

**Keywords:** azo materials, azo glass, directional photo-fluidization, reversible photo-induced reshaping, stimuli-sensitive structures, nanoimprint lithography

## Abstract

A blend of low molecular azo glass (AZOPD) and polystyrene (PS) were used for the systematic investigation of photo-induced stretching and recovery of nanoimprinted structures. For this purpose, light and heat was used as recovery stimuli. The AZOPD/PS microstructures, fabricated with thermal nanoimprint lithography (tNIL), comprises three different shapes (circles, crosses and squares) and various concentrations of AZOPD fractions. The results show a concentration-dependent reshaping. Particularly the sample with 43 w-% of the AZOPD fraction have shown the best controllable recovery for the used parameters. A possible explanation for shape recovery might be the stabilizing effect of the PS-matrix.

## 1. Introduction

For several decades, manufacturing methods have been further developed to fabricate micro- and nanostructures cost- and time-efficiently, while maintaining high precision in shape and in high throughput [1,2,3]. Ruprecht et al. demonstrated a high aspect ratio microstructuring using LIGA process (German acronym for lithography, electroplating, and molding) already in 1993 [4]. Two years later, Chou et al. published for the first time on thermal nanoimprint lithography process (tNIL) showing sub-25 nm structures [5,6,7]. Since then it has been complemented by different techniques (e.g., thermal NIL, reverse NIL, UV NIL, combination of NIL with photolithography) [3,8,9,10,11,12,13] to fabricate programmable and functional micro- and nanoparticles [14,15]. One important advantage of this method is the wide range of materials that can be used depending on the desired application, e.g., biodegradable materials for drug delivery [16,17], stimuli-responsive materials for optical systems, sensor technology, etc. [9,18,19,20,21].

A challenging but important ability for those applications is represented by reversible deformation of micro- and nanostructures with following access to initial geometry. In particular, photo-induced reversible deformation enables a wide range of applications, especially in actuators [22,23], soft and micro robotics [23,24], photo-switches [25,26,27] and surface functionalization [28,29,30]. Probably the first work on reversible photo-induced deformation of an azo dye containing material was done by Merian in 1966. Here, different tissues colored with azo dyes were shrinked after illumination with a daylight lamp. The initial geometry was recovered in darkness [31]. About 40 years later, the anisotropic light-driven stretching of colloids made of side-chain azo polymers was performed by the group of Xiaogong Wang probably for the first time. The stretching was achieved by irradiation with a polarized and monochromatic single laser beam [32,33,34,35]. It could be demonstrated by several groups that the photo-induced stretching could be recovered to the initial geometry by irradiating the same area again with perpendicularly polarized light (compared to first irradiation) [36,37,38]. To name but a few examples, Wang et al. [36] demonstrated photo-induced recovery to the initial shape on breath figure arrays using an azo polymer, Ichikawa et al. [37] have done the same on irregularly shaped particles of low-molecular azo compounds (azo glasses) embedded in agar gel, and Pirani et al. [38] have used imprinted micro-pillars consisting of a mixture of an azo polymer and poly(methyl methacrylate) (PMMA) for reversible reshaping. It is noteworthy that the above mentioned groups demonstrated shape change and recovery by alternating the laser polarization of incident light. Furthermore, the important work of Ryabchun et al. shows shape recovery upon irradiation with non-polarized UV-light or annealing above glass transition temperature ($T_{g}$) after stretching spherical particles of azo polymers in a blend of liquid-crystalline side-chain azo copolymer and an elastomeric triblock copolymer with linear polarized UV-light [39].

The major research on directional stretching of micro- and nanostructures containing azo materials has been done with azo polymers because of their diverse properties ranging from amorphous to liquid crystalline features [15,18,28,32,33,35,36,38,39,40,41,42,43,44,45,46]. However, it was shown that low molecular azo glasses are good candidates for directional photo-induced deformation, too [37,47,48,49,50,51]. In comparison to polymers, the synthesis of azo glasses allows an advantageous control for uniform molecular weight. In addition to an easier purification after synthesis, a good utilization and reproducibility of thermodynamic properties like $T_{g}$ is possible [52,53]. A very interesting molecule for this work is the low molecular azo glass named *N,N’*-bis(phenyl)-*N,N’*-bis((4-phenylazo)-phenyl)benzidine (AZOPD), which can be used for photo-induced structuring for holographic data storage devices [52]. Because of its extraordinary electrochemical properties it can be considered as a multifunctional organic semiconductor (e.g., as an hole transporting material in organic light emitting devices (OLED) or as a photo-switch in organic transistors) [54,55].

**Figure 1:**
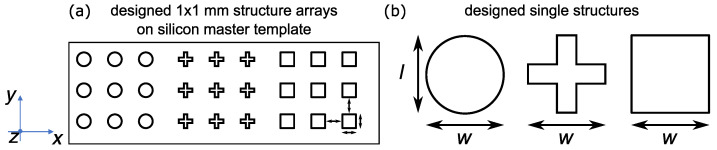
Schematic representation of the structures (circles, crosses and squares) on the silicon master template. The width *w* and length *l* of the structures and the spacing were each designed to be 3 µm long in an array over 1 × 1 mm. Each arrow is 3 µm in length.

In this work, we present reversible photo-induced stretching of microstructures fabricated via tNIL consisting of a blend of the self-synthesized low molecular glass AZOPD as the chromophoric part and polystyrene (PS). Such a dimeric molecule as AZOPD has a low tendency of crystallization and, therefore, an amorphous nature can be obtained easily. The structured films were prepared to be in the amorphous phase by molding the dropcasted AZOPD/PS mixture. Photo-induced stretching was performed using a horizontally polarized (along *x*-axis in Figure 1) laser beam, whereas the recovery of structures was performed with either a second exposure by stretching in perpendicular direction or annealing the sample above $T_{g}$. In order to evaluate the tunability, the reversible photo-induced stretching and recovery via light and heat were performed systematically. For this purpose, the reversibility experiments were done with three different fractions of AZOPD for three different imprinted microstructure geometries and compared with one another.

## 2. Materials and Methods

### 2.1. Fabrication of the PDMS Stamp

To fabricate the silicon master template, e-beam lithography (eLine Plus, *Raith*, Germany) was used. For this purpose, circular, cross, and square shaped structures with a width *w*, length *l* and spacing of 3 μm each was designed (see Figure 1). The polydimetylsiloxane (PDMS) stamp was fabricated as a hybrid stamp comprising hard-PDMS (h-PDMS) and soft-PDMS (s-PDMS) on a glass carrier. h-PDMS is prepared by mixing component A (100 g vinylmethylsiloxane–dimethylsiloxane trimethylsiloxy-terminated copolymer (AB112958), 0.38 g platinum–divinyltetramethyldisiloxane complex (AB153234) and 1.27 g 1,3,5,7-tetravinyl-1,3,5,7-tetramethylcylotetrasiloxane (AB109175) purchased from ABCR GmbH) and B (methylhydrosiloxane–dimethylsiloxane copolymer (AB109380), purchased from ABCR GmbH)) in a weight ratio of 3:1. The mixture was spin-coated for 20 s at 1000 rpm on the silicon master. After drying for 20 min at 65 ${}^{\circ }$C, s-PDMS was drop casted onto h-PDMS and covered with the pre-cleaned glass carrier. s-PDMS is prepared by mixing the base and curing agent SYLGARD^TM^ 184 (purchased from *Dow Corning*) in a weight ratio of 10:1. Afterward, the construction was cured overnight at 65 ${}^{\circ }$C. The silicon master template can separated gently and the hybrid mold can be used for imprinting [9,10].

### 2.2. Fabrication of Structured AZOPD-PS Film

**Figure 2:**
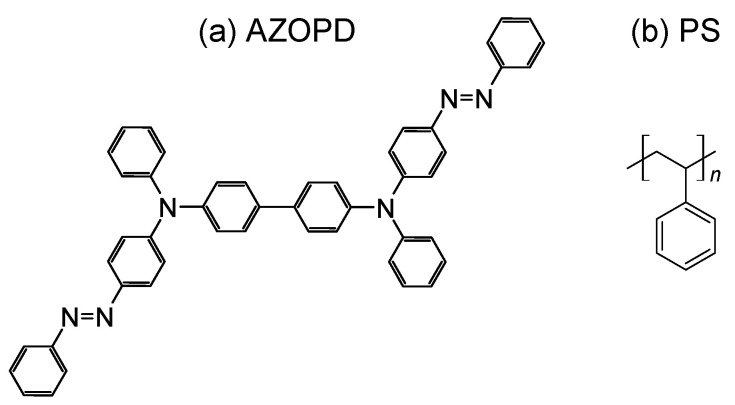
Chemical structures of (**a**) AZOPD and (**b**) polystyrene.

**Figure A1:**
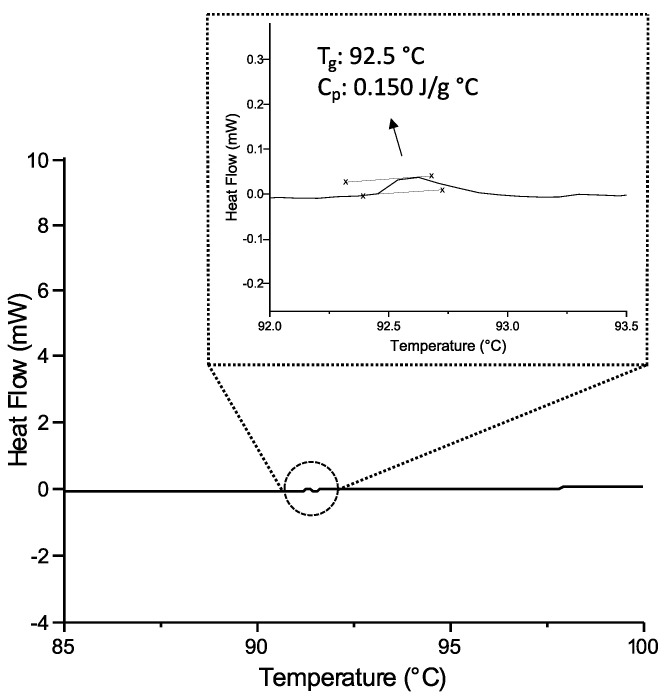
DSC measurement with the Azo30 film. The fitted range at the $T_{g}$-step is magnified and fitted.

To investigate the stretching behavior, a structured AZOPD-PS film was fabricated. The synthesis of AZOPD is reported elsewhere [52]. For film fabrication, AZOPD (Figure 2a) and PS ($\bar{M_{w}}\approx 280,000$ by GPC, $T_{g}=100{\;}^{\circ }$C, purchased from Sigma Aldrich, St. Louis, MO, USA, Figure 2b) were solved in tetrahydrofuran (THF) each. The $T_{g}$ values for each mixture were found out by measuring with a differential scanning calorimeter (DSC, PerkinElmer DSC 7, Waltham, MA, USA) in the frame of a thermal profile from 40 ${}^{\circ }$C to 150 ${}^{\circ }$C with a heating rate of 5 ${}^{\circ }$C/min under nitrogen atmosphere (90 mL/min). The measurement resulted in a glass transition temperature of about $T_{g}\;\approx \;93{\;}^{\circ }$C for each mixture (see Figure A1). This is close to the $T_{g}$ of each component (AZOPD: $T_{g}\;=\;101{\;}^{\circ }$C [52]. and PS: $T_{g}=100{\;}^{\circ }$C (according Sigma Aldrich)).

**Figure 3:**
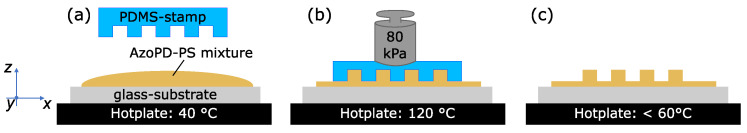
Schematic representation of structuring the AZOPD-PS film via tNIL. (**a**) 300 µL of an AZOPD-PS solution was dropcasted on a glass substrate and heated at 40 ${}^{\circ }$C for 30 s to remove partly the solvent. (**b**) The PDMS stamp was placed onto the AZOPD-PS layer and a pressure of 80 kPa was applied for 30 min at 120 ${}^{\circ }$C. (**c**) After cooling below 60 ${}^{\circ }$C the PDMS stamp was separated.

Solutions of AZOPD with c=30mg/mL and PS with c=40mg/mL were mixed in a ratio of 1:1 and stirred for 24 h to guarantee a homogeneous mixture. The solution with this AZOPD concentration and the respective film, which has an amount of 43 w-% of the AZOPD fraction, will be called Azo30. Analogously, a solution of AZOPD 10 mg/mL and PS 40 mg/mL (Azo10) and AZOPD 50 mg/mL and PS 40 mg/mL (Azo50) were prepared and mixed with the same ratio. The respective films of Azo10 (20 w-% of AZOPD fraction) and Azo 50 (56 w-% of AZOPD fraction) will be called equally. The glass substrate is pre-cleaned with bidest. water, acetone and with isopropyl alcohol afterwards. 300 µL of each AZOPD-PS-solution was drop casted on the pre-cleaned substrate. Then the coated substrate was heated at 40 ${}^{\circ }$C for 30 s to evaporate the major part of the solvent. The PDMS stamp was placed onto the AZOPD-PS film. Pressure was applied on top of the PDMS stamp to ensure a homogeneous contact between the AZOPD-PS layer and the PDMS stamp (see Figure 3). Afterwards, the construction was heated at 120 ${}^{\circ }$C for 30 min, which is above the $T_{g}$ of the mixture (Figure 3b). After annealing, the sample was cooled down slowly below 60 ${}^{\circ }$C and the PDMS stamp was separated. Finally, the structures are transferred onto the AZOPD-PS film (Figure 3c).

### 2.3. Actuation

**Figure 4:**
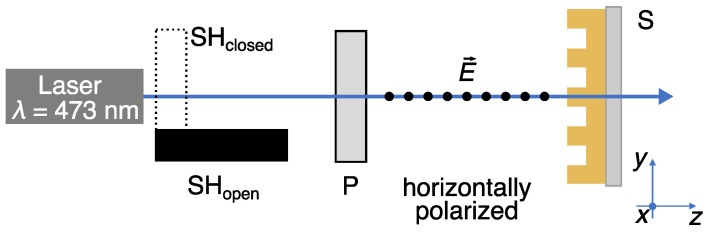
Schematic draw of the experimental setup. The linear polarization orientation $\vec{E}$ along *x*-axis is ensured by a polarization filter **P**. The sample **S** was placed into a movable sample holder to align it in *x-y*-plane, which is perpendicular to the beam propagation (*z*-axis). The automated shutter **SH** was placed in front of the laser to block the laser beam after the illumination duration.

For stretching the structures on the AZOPD-PS film, linearly polarized light was used (DPSS-laser, 473 nm, fluence ≈1200 mW/cm${}^{2}$) as shown in Figure 4. This wavelength addresses either, *cis*- and *trans*-configuration, which results in a repetitive isomerization behavior (photo-orientation). The duration of irradiation was controlled by using an electronic shutter in front of the laser. The linear polarization orientation $\vec{E}$ of the laser beam along *x*-axis is ensured by a polarization filter. The recovery of unstretched structures was done either by relaxing with thermal treatment on a hotplate at 120 ${}^{\circ }$C for 5 min or by photo-induced restretching along *l*-direction (perpendicular to first stretching axis) for 10 min. The spots for the different actuation and reactuation steps were marked and the sample is placed at a moveable sample holder to ensure the same placement of the sample for each treatment step.

### 2.4. Measurement

**Figure 5:**
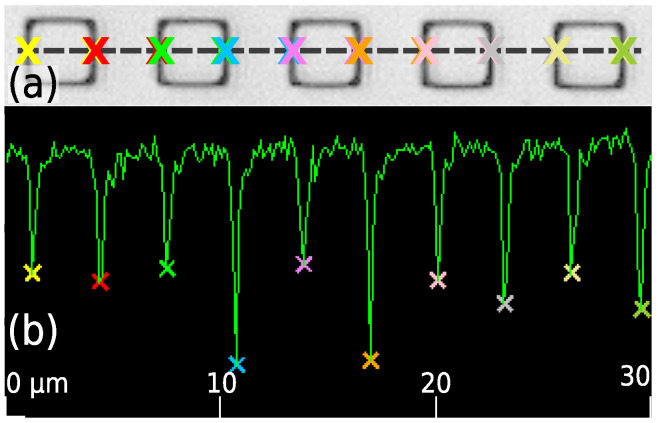
Exemplary images from the auto-measurement tool. (**a**) A laser micrograph of imprinted square structures with a drawn baseline to acquire the intensity profile. (**b**) Resulted intensity profile along baseline with marked intensity peaks as colored crosses to determine the *w* and *l* values.

Displacement measurements were performed using a confocal laser scanning microscope (VK-X1100, *Keyence*). The samples were neither affected by the laser beam of the VK-X1100 nor by daylight. Using the company provided VK-Analyzer, the intensity profile was acquired by using a baseline along *w*- or *l*-direction. By using the auto-measurement tool, the absolute values of *w* and *l* lengths of more than 150 single structures were measured for each treatment step. As an example, the laser image of nanoimprinted structures (Figure 5a) and the intensity profile (Figure 5b) along the baseline (dashed line) is shown. The absolute *w* values were determined from the distance between the intensity peaks (colored crosses). The *l* values were determined analogously. With a self written Python program the mean average for the *w* and *l* values within a confidential interval of 0.95 were determined.

## 3. Results

For the reversal of anisotropic photo-induced stretching two different stimuli is investigated in this paper: the first one is the recovery by restreching via linearly polarized light (perpendicular to first irradiation) and the second one is the recovery by relaxing via heating above $T_{g}$. For each method, two cycles of stretching and recovery were performed for each structure (circles, crosses, and squares). At first, the structures were stretched along *w*-direction in all cases. In method 1, the light-induced restretching was accomplished in *l*-direction (Figure 1), whereas in method 2 no direction can be defined for relaxing to the initial geometry upon heating.

Since the structures are symmetrical but of different shape, only the w/l-ratio has been taken into account for the evaluation. The relative ratio w/l was calculated from the absolute *w* and *l* average values (determined from more than 150 single structure) for each treatment step (see Appendix B Table A2 and Table A3). In the following, the dependency between the w/l-ratio of an Azo30 sample and the time of illuminating the structures will be shown. Subsequently, the reversibility of stretching and recovery by light or temperature is presented for Azo10, Azo30, and Azo50 samples.

### 3.1. Time Dependency of Stretching Microstructures

For further investigations samples with medium AZOPD concentration (Azo30) were selected in order to set the duration for illuminating the samples. The illumination duration of 2, 10, and 30 min were chosen for the Azo30 sample based on previous report [56].

**Figure 6:**
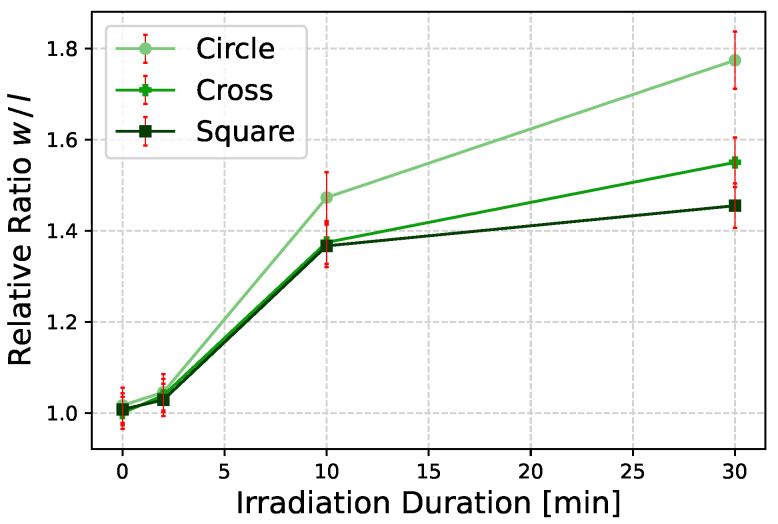
Relative w/l-ratio as a function of irradiation duration for Azo30. An increase in size can be observed for circle and cross structures at longer illumination, whereas the increase in size for square structures is negligible after 10 min.

There was no significant change in the *w* nor in the *l* size for all three structures while the sample was exposed for 2 min. Upon a longer illumination time of 10 min all structures exhibit an increase of around 25% in size along the *w*-direction compared to their untreated size. After 30 min of irradiation, the circle structures were stretched nearly 60% in *w*-direction in comparison to the untreated size, whereby cross structures show an elongation of 38%. In contrast to the circle and cross, the square structures show a gain of 27 % in size after 30 min, compared to initial structure. As can be seen in the diagram (Figure 6), the elongation quantity for an illumination of 10 and 30 min is nearly the same.

**Figure 7:**
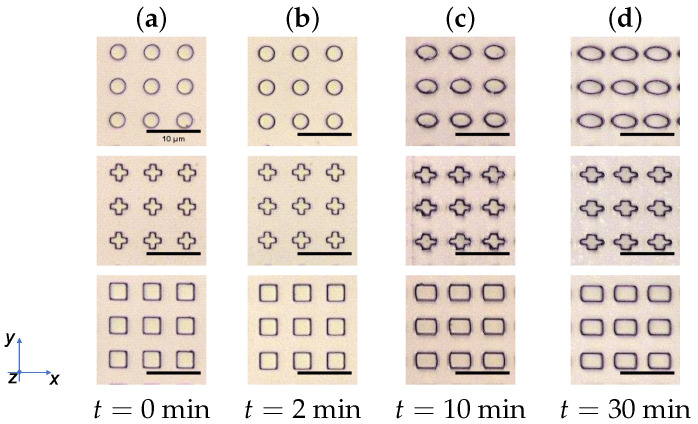
Micrographs of treated Azo30 structures. (**a**) Structures after the fabrication process with tNIL. (**b**) After 2 min of illumination with horizontally polarized light, a slight change can be measured. (**c**) Upon 10 min, a clear stretching in *w*-direction was measured. (**d**) Larger stretching is obtained after an illumination for 30 min.

While the *w* size increases upon illumination for 10 min, the *l* size shrinks by 10% for circle and square and 8% for the cross structures. On the other hand, the square structures show a negligible change in the *w* and *l*-direction comparing 10 min to 30 min of illumination. However, for the elongation in *l*-direction the circle and cross shaped structures shrink up to 18% after an illumination of 30 min. Due to the comparability, an irradiation duration of 10 min was selected for further investigation because a similar stretching ratio results at this point. Micrographs in Figure 7 visualize treated Azo30 structures after each illumination duration.

### 3.2. Reversible Change of the Structure Sizes by Light

Based on the results in Section 3.1, an irradiation duration of 10 min was chosen as appropriate for each reshaping step. To investigate these behavior, the untreated samples (designated as L0; “L” for light-induced recovery) were illuminated with horizontally polarized light for 10 min in the *w*-direction during the first treatment step (L1). The second treatment (L2) is done analogously in *l*-direction. Subsequently, a second cycle with treatment L3 and L4 were performed as in L1 and L2.

**Figure 8:**
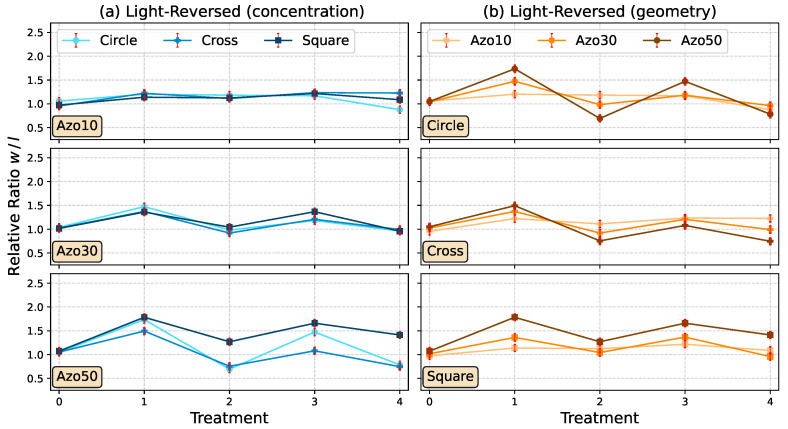
The ordinate shows the relative w/l-ratio of the mean values corresponding to different concentrations (Azo10, Azo30 and Azo50) in (**a**) and each geometry (circle, square and cross) in (**b**). The abscissa shows the treatment steps (L0: untreated, L1: stretching for 10 min in *w*-direction, L2: stretching for 10 min in *l*-direction, L3: same as L1, L4: same as L2.

**Figure 9:**
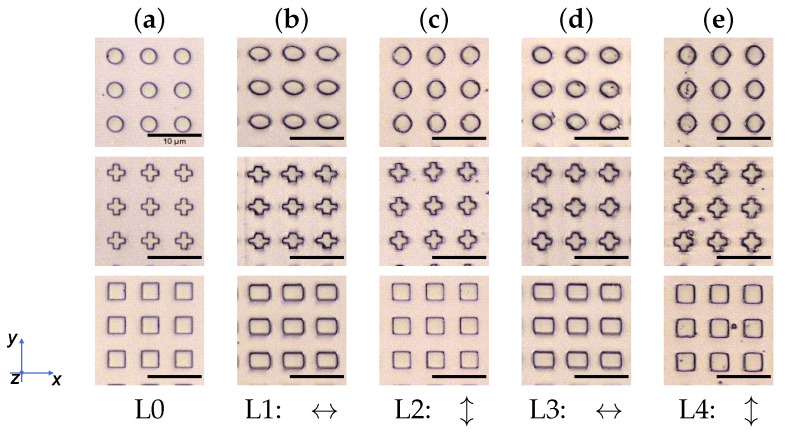
Micrographes of treated Azo30 structures. (**a**) Structures after the fabrication process with tNIL. (**b**) All three structures get stretched after the first treatment in the *w*-size while the *l*-size shrinks. (**c**) After L2, the *w*-size shrinks again while the *l*-size elongates. (**d**) By repeating the stretching in *w*-direction (L3), the structures increase in size again. (**e**) In L4, the structures are mainly recovered after restretching along *l*-direction.

While there is no significant reversible stretching measurable for the Azo10 samples (Figure 8a, top), the Azo30 (a, middle) and Azo50 (a, bottom) samples show a clear behavior in stretching and restretching to the initial size. During treatment L3, the structures can be stretched by 20%, while for treatment L4 the initial geometry could be restored as it can be seen in Figure 8. Azo50 seems to be unsuitable for a controlled recovery of the initial size. Additionally, by illuminating the structures in step L2, the *l*-size extends while the *w*-size decreases for circle and cross. For square structures, the second treatment L2 seems insufficient to recover the structures completely. Irradiation during treatment L3 leads again to an increase in *w*-size up to 23% for cross and square and around 55% for circle structures. The treatment step L4 results in a similar behavior as in L2, whereby for all three structures the sizes remain similar. Even though, the structure types behave different in their reshaping, there is no clear geometry dependency visible for all samples (Figure 8b). Micrographs in Figure 9 visualize Azo30 structures after each treatment step.

### 3.3. Reversible Change of the Structure Sizes by Temperature

**Figure 10:**
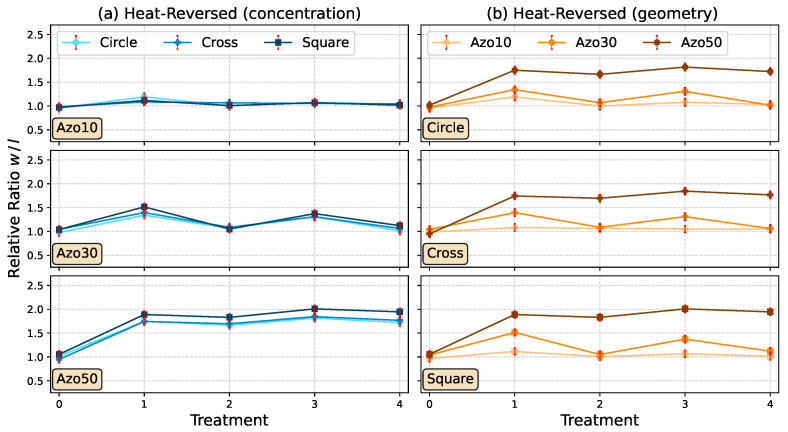
The ordinate shows the relative w/l-ratio of the mean values considering each concentration (Azo10, Azo30 and Azo50) in (**a**) and each geometry (circles, crosses and squares) in (**b**). The abscissa shows the treatment steps (T0: untreated, T1: stretching for 10 min in *w*-direction, T2: heating at 120 °C for 5 min, T3: same as T1, T4: same as T2).

**Figure 11:**
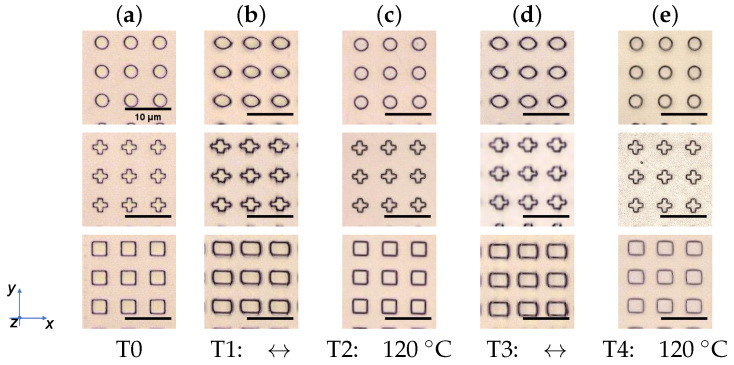
Micrographes of treated Azo30 structures. (**a**) Structures after the fabrication process with tNIL. (**b**) All three structures get stretched after the first treatment in the *w*-size while the *l*-size shrinks. (**c**) During T2, the *w* and *l*-sizes relax. (**d**) By repeating the stretching in *w*-direction (T3), the structures increase in size again. (**e**) During T4, the structures are mainly recovered after relaxing.

As already mentioned, the light-induced stretching can additionally be relaxed by means of temperature. Here, treatment steps T0, T1 and T3 (“T” for thermal-induced recovery) remain the same as for L0, L1, and L3, but treatment steps T2 and T4 are replaced by a temperature step. During these steps, the samples were heated at 120 ${}^{\circ }$C for 5 min to relax the stretched structures to the initial size. Again, the investigation of the reversibility results in a dependency on AZOPD-concentration (Figure 10). Micrographs in Figure 11 visualize Azo30 structures after each treatment step.

The results of the Azo10 sample (Figure 10a, top) show no significant change in the structures for both stretching and relaxing (below 10%). In contrast, the Azo30 sample (a, middle) shows a clear stretching (T1 and T3) and relaxation in T2 and T4 about 25% of the initial size. For the Azo50 sample (a, bottom), the first stretching with light is irreversibly high (approx. 70%). It was not possible to relax the stretched structures to initial size after 5 min of heating during T2 (nearly 45% remains). Even after the second cycle, the initial size could not be recovered. However, a slight stretching was still possible during T3.

The calculated w/l-ratio of square structures is higher for Azo50 samples than for circle or cross structures (best visible after T1 in Figure 10a, middle and bottom). However, likewise as light-reversed shape deformation, it is not possible to identify a significant dependency for the geometries (Figure 10b).

## 4. Discussion

The experiments demonstrate that the reversible stretching of imprinted structures, made out of an AZOPD/PS-mixture, can be implemented by a second stretching along *l*-direction or relaxing by annealing above $T_{g}$. The reversible shape deformation was performed twice to recover the initial structure geometry. Depending on irradiation duration and the concentration of the AZOPD fraction, the relative stretching quantity ranges between 10–70%, which is comparable with results from literature. Comparable experiments show relative stretching ranges between 30–140% [38,39,48,49,50]. However, a small degree of degradation was obtained after the second reversion, which is observable in a decrease in the w/l-ratio. Findings of Pirani et al. show a similar degradation effect [38]. A possible explanation is that a small amount of AZOPD and/or PS remain in the stretched orientation and are not able to be addressed.

Comparing the light- and heat-treated reversible reshaping, it is visible that the recovery of initial geometry is less deviating with heat-reversion (see micrographs in Figure 9 and Figure 11). This effect can be understood when it is taken into account that thermal reversal is accomplished only by relaxing the stretched structure, whereas light-reversed structures experience an additional perpendicular stretching in *l*-direction (as seen in Figure 9). Consequently, light-recovered structures are not only relaxed, but also stretched, whereas heat-recovered structures are only relaxed to initial geometry. For better results in recovery by polarized light, the duration of irradiation should be readjusted, i.e., the structures should not be irradiated in *l*-direction as long as in *w*-direction. The *l*-direction needs shorter irradiation duration to prevent an unintended *l*-stretching after the first treatment in *w*-direction. In contrast, the thermal recovery at the chosen temperature prevents an additional stretching and the last achievable condition is the initial geometry. Another observation is that the amplification of the stretching effect with higher AZOPD concentration does not behave in a linear way as seen in Figure 8 and Figure 10. A five-fold AZOPD concentration between Azo10 and Azo50 does not result in a five-fold higher stretching value. Therefore, it is suspected that the stretching quantity behaves similar to the stretching behavior itself, namely with a fast beginning increase and ending in reaching slowly a saturation point. This behavior is very similar to well known photo-induced surface and bulk structuring of azo films [56,57,58,59].

The mechanism of stretching can be divided into three different steps: (1) photo-isomerization, (2) photo-orientation, and (3) photo-induced mass transport/flow. The step (1) of photo-isomerization is the fastest step and describes the *trans-cis*-isomerization, whereas the second step (2) of photo-orientation occurs under the condition of constantly irradiating the azo material with polarized light with a wavelength within the n-π*-absorption. Here, the isomerization has a repetitive behavior and azo molecules undergo a cyclic *trans-cis-trans*-isomerization since either configurations are addressed [57,60]. The probability of isomerization *W* decreases, when the angle θ between the polarization plane, e.g., the E-field vector of polarized light, and the dipole moment of the azo molecule tends to 90${}^{\circ }$: $W\propto \cos^{2}\theta$ [57,60]. Once the dipole moment of the azo molecule and the polarization orientation of incident light is perpendicular to each other, photo-isomerization does not occur anymore. Afterwards, the third step (3) of directional photo-induced mass flow along the polarization orientation starts. It can be assumed that the transition between each step is continuous. At this step, azo molecules move or flow parallel to the polarization plane. This process is temporally limited and stops after reaching a saturation point [27,34,37,39,46,47,56,57]. As can be seen in Figure 6, the time dependency of light-induced stretching can be confirmed.

An interesting idea for the mechanism is described by the group of Hideyuki Nakano [37,47]. They describe the elongation process of azo particles in agar-gel as a push-pull effect between particles of the azo glass and surrounding agar-gel. Ryabchun et al. made a similar assumption. Our results may support the hypothesis that recovery of a deformed structure to initial geometry is highly supported by the elastic polymer matrix, which is embedding the azo material. The AZOPD material within the PS-matrix undergoes photo-induced stretching (mechanical strain), which in turn, stretches the polymer matrix of PS and keeps it under stress. Upon annealing above $T_{g}$, the polymer matrix of PS relaxes and compresses the stretched AZOPD material. A hint for this is that the results presented here show a significant increasing of the stretching behavior with higher relative proportion of AZOPD in the AZOPD/PS-mixture. It is reasonable that a higher concentration of chromophore can apply a bigger force on the surrounding polymer matrix during stretching, which leads to higher degree of relative stretching and restretching. However, systematic experiments need to be done with various molecular weights of PS.

## 5. Conclusions

Reversible reshaping of imprinted periodic structures by using linearly polarized light or heat as the stimuli for recovery has been shown successfully. The dependency on AZOPD-concentration and structure geometry for stretching and restretching/relaxing were investigated in a systematic way for either recovery-stimuli. A good controllable stretching can be achieved with a specific concentration ratio, which is represented by Azo30 samples. The results can be applied for functional structures produced by means of diverse NIL-techniques. Another application is to use the cross-structure as a light-driven switch in electronic micro- or nano-circuits by stretching the opposite cross-arms toward or away a circuit, whereas an orthogonal stretching would address a second circuit. However, the reaction time of photo-induced stretching has to be improved significantly for electronic circuits.

## Data Availability

The datasets used and/or analyzed during the current study are available from the corresponding author on reasonable request, including the used python code.

## Abbreviations

The following abbreviations are used in this manuscript:

AZOPD	*N,N’*-bis(phenyl)-*N,N’*-bis((4-phenylazo)-phenyl)benzidine
PS	Polystyrene
tNIL	Thermal nanoimprint lithography
PMMA	Poly(methyl methacrylate)
$T_{g}$	Glass transition temperature
OLED	Organic light emitting device
PDMS	Polydimetylsiloxane
THF	Tetrahydrofuran
DSC	Differential scanning calorimetry
DPSS	Diode pumped solid state
*W*	Probability of isomerization

## Appendix A. DSC-Measurement of Azo30

The $T_{g}$ values for the mixtures are close to one another. Exemplary, the heat flow curve of the Azo30 mixture is shown here. The resulted glass transition temperature from the second run is $T_{g}=92.5{\;}^{\circ }$C with $C_{p}=0.15$ J/g ${}^{\circ }$C.

## Appendix B. Absolute Values of Widths and Lengths

### Appendix B.1. w/l Values for Time Depending Stretching

**Table A1:** Average *w* and *l*-values and their standard deviation for different irradiation duration for the Azo30 sample.

Shape	Irradiation Duration [min]	$w_{\mathrm{average}}$ [µm]	$w_{\mathrm{std}}$ [µm]	$l_{\mathrm{average}}$ [µm]	$l_{\mathrm{std}}$ [µm]
Circle	0	2.819	0.075	2.776	0.075
	2	2.835	0.075	2.712	0.075
	10	3.613	0.076	2.453	0.076
	30	4.340	0.076	2.446	0.075
Cross	0	3.019	0.076	3.022	0.075
	2	3.090	0.074	2.975	0.076
	10	3.773	0.077	2.745	0.076
	30	4.046	0.080	2.610	0.076
Square	0	3.069	0.075	3.047	0.075
	2	3.091	0.075	3.004	0.075
	10	3.762	0.076	2.752	0.076
	30	4.023	0.076	2.765	0.076

### Appendix B.2. w/l Values for Light and Heat-Reversed Stretching

**Table A2:** Average *w* and *l*-values and their standard deviation for light-reversed tunability.

Treatment	Sample	$w_{\mathrm{average}}$ [µm]	$w_{\mathrm{std}}$ [µm]	$l_{\mathrm{average}}$ [µm]	$l_{\mathrm{std}}$ [µm]
0		2.771	0.076	2.622	0.076
1	Azo10	3.118	0.077	2.592	0.076
2	Circle	3.063	0.076	2.592	0.076
3		3.034	0.076	2.596	0.076
4		2.656	0.075	3.028	0.076
0		2.852	0.075	2.743	0.076
1	Azo30	3.611	0.076	2.451	0.076
2	Circle	3.024	0.076	3.076	0.075
3		3.337	0.077	2.825	0.079
4		3.024	0.077	3.142	0.078
0		2.771	0.076	2.644	0.076
1	Azo50	4.032	0.077	2.324	0.076
2	Circle	2.890	0.076	4.167	0.076
3		4.467	0.077	3.033	0.076
4		3.396	0.080	4.330	0.076
0		2.916	0.077	3.053	0.077
1	Azo10	3.388	0.076	2.772	0.076
2	Cross	3.232	0.075	2.907	0.076
3		3.461	0.076	2.802	0.075
4		3.435	0.075	2.800	0.075
0		3.069	0.075	2.997	0.075
1	Azo30	3.783	0.077	2.755	0.076
2	Cross	3.029	0.076	3.294	0.077
3		3.712	0.079	3.074	0.076
4		3.438	0.078	3.466	0.076
0		3.080	0.075	2.928	0.075
1	Azo50	3.914	0.077	2.620	0.076
2	Cross	3.030	0.076	4.013	0.076
3		3.738	0.078	3.463	0.076
4		3.052	0.077	4.089	0.074
0		2.955	0.074	3.038	0.076
1	Azo10	3.325	0.076	2.919	0.076
2	Square	3.279	0.075	2.918	0.075
3		3.468	0.076	2.849	0.074
4		3.320	0.076	3.055	0.076
0		3.071	0.075	3.022	0.075
1	Azo30	3.760	0.076	2.765	0.077
2	Square	3.168	0.076	3.038	0.076
3		3.740	0.076	2.736	0.076
4		3.204	0.076	3.342	0.076
0		3.150	0.075	2.938	0.075
1	Azo50	4.756	0.076	2.667	0.074
2	Square	4.204	0.076	3.312	0.076
3		4.742	0.078	2.858	0.075
4		4.434	0.076	3.140	0.075

**Table A3:** Average *w* and *l*-values and their standard deviation for thermal-reversed tunability.

Treatment	Sample	$w_{\mathrm{average}}$ [µm]	$w_{\mathrm{std}}$ [µm]	$l_{\mathrm{average}}$ [µm]	$l_{\mathrm{std}}$ [µm]
0		2.615	0.076	2.739	0.076
1	Azo10	3.033	0.076	2.545	0.077
2	Circle	2.623	0.076	2.627	0.076
3		2.829	0.077	2.627	0.076
4		2.623	0.076	2.543	0.078
0		2.683	0.076	2.750	0.076
1	Azo30	3.301	0.076	2.460	0.076
2	Circle	2.788	0.075	2.609	0.076
3		3.407	0.077	2.606	0.076
4		2.683	0.076	2.647	0.076
0		2.777	0.076	2.737	0.076
1	Azo50	4.252	0.079	2.430	0.075
2	Circle	4.124	0.078	2.480	0.076
3		4.382	0.076	2.414	0.076
4		4.207	0.076	2.442	0.075
0		2.897	0.076	2.940	0.075
1	Azo10	3.072	0.076	2.835	0.077
2	Cross	2.975	0.075	2.794	0.075
3		2.950	0.074	2.807	0.074
4		2.782	0.078	2.653	0.076
0		3.021	0.076	2.895	0.076
1	Azo30	3.733	0.082	2.675	0.078
2	Cross	3.068	0.076	2.823	0.078
3		3.615	0.076	2.761	0.077
4		2.999	0.075	2.823	0.077
0		2.777	0.076	2.927	0.075
1	Azo50	4.587	0.076	2.630	0.076
2	Cross	4.568	0.076	2.692	0.077
3		4.874	0.076	2.640	0.076
4		4.631	0.079	2.621	0.076
0		3.042	0.076	3.140	0.075
1	Azo10	3.312	0.076	2.970	0.075
2	Square	3.065	0.075	3.042	0.076
3		3.196	0.076	2.995	0.075
4		3.018	0.075	2.970	0.077
0		3.073	0.075	2.956	0.074
1	Azo30	4.027	0.076	2.658	0.075
2	Square	3.037	0.076	2.892	0.076
3		4.004	0.075	2.914	0.076
4		3.285	0.075	2.929	0.075
0		3.095	0.074	2.935	0.075
1	Azo50	5.155	0.076	2.727	0.075
2	Square	5.013	0.076	2.739	0.076
3		5.606	0.076	2.791	0.075
4		5.317	0.090	2.733	0.075
